# Metastatic Orbital Tumor From Breast Ductal Carcinoma With Neuroendocrine Differentiation Initially Presenting as Ocular Symptoms: A Case Report and Literature Review

**DOI:** 10.3389/fendo.2021.625663

**Published:** 2021-02-22

**Authors:** Keita Togashi, Koichi Nishitsuka, Shion Hayashi, Hiroyuki Namba, Sakiko Goto, Yusuke Takeda, Shuhei Suzuki, Tomoya Kato, Yuki Yamada, Eriko Konno, Takashi Yoshioka, Mitsunori Yamakawa, Yukihiko Sonoda, Tamio Suzuki, Hidetoshi Yamashita

**Affiliations:** ^1^ Department of Ophthalmology and Visual Sciences, Yamagata University Faculty of Medicine, Yamagata, Japan; ^2^ Department of Clinical Oncology, Yamagata University Faculty of Medicine, Yamagata, Japan; ^3^ Department of Pathology, Fukushima Rosai Hospital, Fukushima, Japan; ^4^ Department of Pathological Diagnostics, Yamagata University Faculty of Medicine, Yamagata, Japan; ^5^ Department of Neurosurgery, Yamagata University Faculty of Medicine, Yamagata, Japan; ^6^ Department of Dermatology, Yamagata University Faculty of Medicine, Yamagata, Japan

**Keywords:** metastatic orbital tumor, breast cancer, neuroendocrine differentiation, cytokeratin 7-negative, visual function

## Abstract

**Background:**

Orbital metastases from cancers of various organs can arise *via* the hematogenous route, and many originate from breast, prostate, and lung cancers. Such metastatic orbital tumors may be diagnosed before the primary tumor. We have encountered a case of breast ductal carcinoma with neuroendocrine differentiation that metastasized to the orbit and responded to chemotherapy, with improvement in visual function.

**Case Presentation:**

A woman in her fifties visited our ophthalmology department with a chief complaint of foreign body sensation and exophthalmos in her right eye. An elastic soft mass was palpated from the lateral orbit to the temporal region. A systemic examination revealed breast cancer and a metastatic orbital tumor. Excisional biopsy of the breast revealed a diagnosis of invasive ductal carcinoma with neuroendocrine differentiation, and immunohistochemical examination was negative for cytokeratin 7, making the case unusual. Chemotherapy was remarkably effective, and the tumor size decreased, resulting in improvement of visual function. Her general condition and quality of life are still good at present. We searched the PubMed English language literature focusing on metastatic orbital tumors from breast cancer in which ocular symptoms had been the initial presenting sign. No previous reports have documented neuroendocrine differentiation or cytokeratin 7 expression in isolated orbital metastases from breast cancer. Although it is not possible to be certain from this case alone, we speculated that some such cases might involve cytokeratin 7-negative invasive breast cancer with neuroendocrine differentiation.

**Conclusion:**

We have described our experience of a very rare case of cytokeratin 7 negative breast ductal carcinoma with neuroendocrine differentiation that metastasized to the orbit and formed a solitary giant tumor initially manifesting as ocular symptoms.

## Introduction

In Japan, malignant lymphoma is the most often responsible for orbital tumors, followed by metastases from cancers of distant organs (1). Orbital metastasis occurs *via* the hematogenous route, and breast, prostate, and lung cancers are most often the primaries responsible (2). Occasionally, metastatic orbital tumors may be diagnosed before the primary lesions. The initial visual impairment in this patient was likely due to the growth of the mass. Therefore, for preservation of visual function and quality of life, prompt diagnosis by an ophthalmologist is essential.

We have encountered a patient with a solitary giant metastatic orbital tumor, complaining of foreign body sensation and exophthalmos in the right eye. A whole-body work-up revealed orbital metastasis from breast cancer. Histopathological examination of the breast cancer showed it to be cytokeratin 7-negative invasive ductal carcinoma with neuroendocrine differentiation. The metastatic orbital tumor responded well to chemotherapy, and as a result, visual function also improved. Orbital metastases from breast cancer often occur as a component of multi-organ metastases. We conducted a literature search focusing specifically on patients with metastatic orbital tumors from breast cancer who had suffered ophthalmologic manifestations as the presenting sign, had no metastases to other organs, and for whom histological diagnoses were available. We then discussed the clinical significance of this case on the basis of clinical and histologic features reported in similar cases.

## Case Presentation

A woman in her fifties presented at the department of ophthalmology in Yamagata University Hospital with hyperemia, lacrimation, foreign body sensation, and exophthalmos in her right eye. She had been aware of the symptoms for four months, and exophthalmos had been developing for two months thereafter. On visiting an ophthalmology clinic at that time, her chief complaints were hyperemia, lacrimation, and foreign body sensation in the right eye. The presence of an orbital tumor was suspected. She denied any history of malignancy.

Initial examination showed that there was no bilateral difference in pupil diameter, and the light reflex was rapid. There was no relative afferent pupillary defect in either eye. Best corrected visual acuity (logMAR unit) was 2.5 (right eye) and 0 (left eye), and intraocular pressure was 22 mmHg (right eye) and 14 mmHg (left eye). Hertel exophthalmometry showed 22 mm (right eye) and 12 mm (left eye). Right exophthalmos was prominent, and an elastic soft mass was palpated from the lateral orbit to the temporal region.

Examination of the anterior segment in the right eye showed superficial punctate keratopathy with erosion below the cornea, and conjunctival injection. Mild cataracts were observed in both eyes. The fundus had no remarkable lesions. Goldmann perimetry showed mildly decreased sensitivity of the central visual field in the right eye. Eye movement of the right eye was limited in superduction.

**Figure 1:**
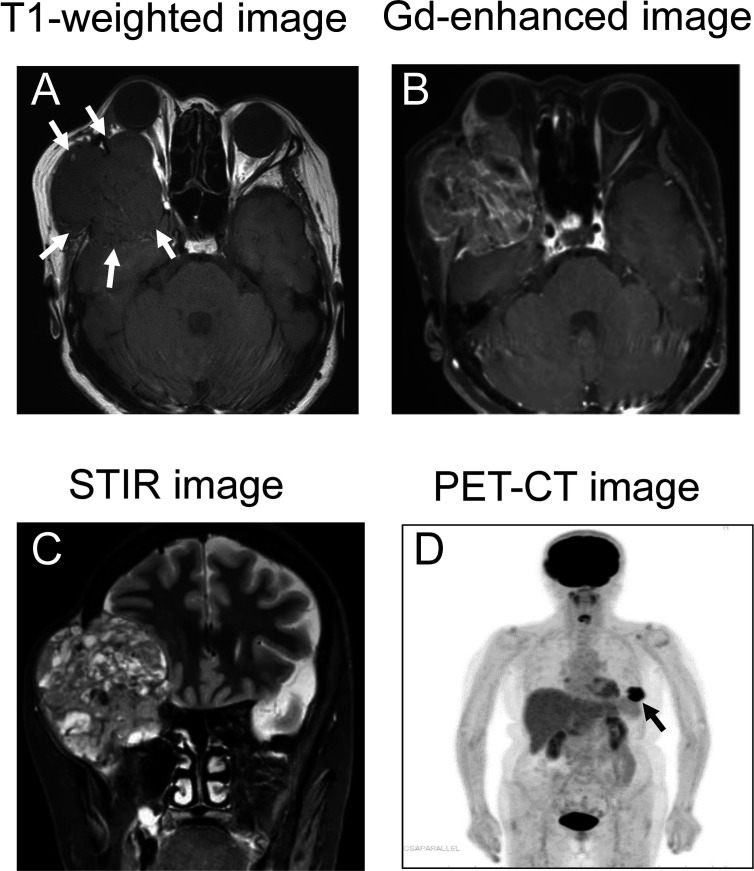
MRI and PET-CT image. **(A)** The tumor displaces the extraocular muscles and optic nerve of the right eye, and brain parenchyma as indicated by arrows (T1-weighted image). Arrows indicate the tumor. **(B)** The tumor showed contrast-enhanced with gadolinium. **(C)** STIR imaging shows a non-homogeneous giant tumor compressing the brain. **(D)** PET-CT demonstrates integration of FDG coincident with the left breast, as shown by the arrow.

MRI revealed a well circumscribed mass lesion measuring 6 × 7.5 × 7 cm behind the right eye. The tumor was contrast-enhancement with gadolinium. Coronal STIR images showed an invasion into extraocular muscles, an optic nerve compression and bone destruction due to a giant mass, and further displacement of the brain parenchyma (**Figures 1A–C**). Multiparametric MRI showed that the orbital tumor was hypointense and hyperintense relative to fat on T1-weighted and T2-weighted images, respectively, as well as being contrast enhanced by gadolinium, thus showing the characteristics of a metastatic tumor. FDG - PET/CT revealed irregular accumulation of FDG in the left breast (**Figure 1D**). There was no significant accumulation of FDG in other organs. The orbital tumor was considered to have metastasized from breast cancer because the breast cancer was diagnosed by PET-CT and MRI. Blood tests for tumor markers, including breast cancer, were negative. Her Eastern Cooperative Oncology Group Performance score was 1.

The patients had been aware of a slow-growing lump in her left breast for several years, but had not sought medical advice. An ulcer measuring 2.0 × 1.8 cm being surrounded with erythematous lesion (2.9 × 2.3 cm) was found in the left breast and we performed an excisional biopsy from the ulcer lesion.

**Figure 2:**
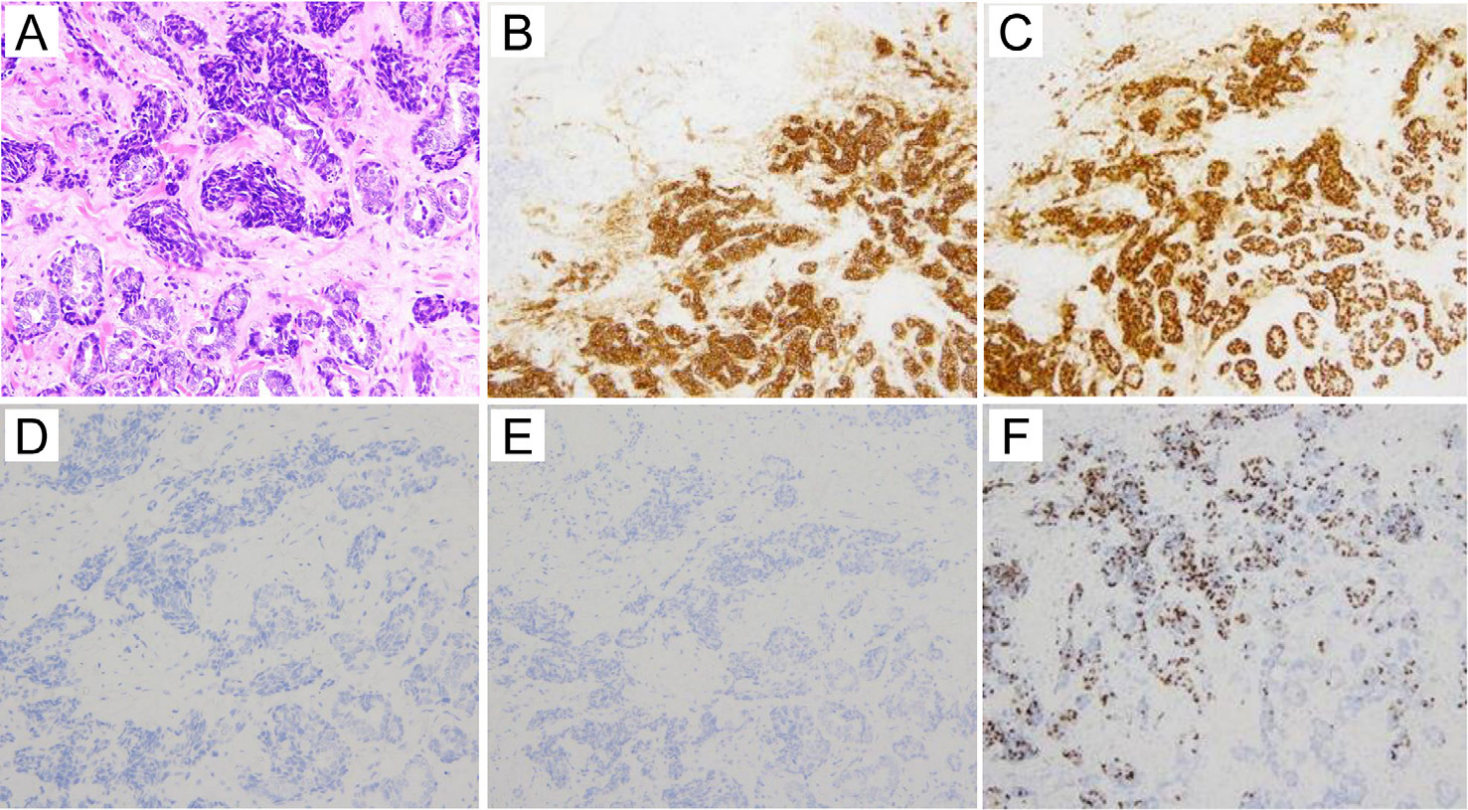
Histological images (HE staining and immunostaining). **(A)** HE staining of the excisional breast biopsy specimen. The tumor cells form tubular structures and solid nests. The tumor cells have small, round to spindle-shaped nuclei with a fine chromatin pattern. (**A**:×20) **(B)** Immunohistologically, synaptophysin is positive (×10). **(C)** Estrogen receptor is strongly positive (×10). **(D)** Cytokeratin 7 is negative (×20). **(E)** Cytokeratin 20 is negative (×10). **(F)** The Ki67 proliferative index is as high as 26% (×10).

The pathology of the left breast ulcer is shown in **Figure 2**. The tumor cells had small, round to spindle-shaped nuclei with a fine chromatin pattern, and formed tubular structures and solid nests. Because the solid nests had features suggestive of carcinoid, several relevant markers were investigated. Synaptophysin was positive, chromogranin A was negative (**Figure 2B**), and neuron-specific enolase was slightly positive (**Supplementary Figure 1**). These findings allowed us to establish a diagnosis of invasive ductal carcinoma with neuroendocrine differentiation. Immunohistochemistry showed negativity for cytokeratin (CK) 7 and CK20 (**Figures 2D, E**), strong positivity for estrogen receptor (**Figure 2B**), positivity for progesterone receptor, and negative for human epidermal growth factor receptor 2 (HER2) (**Supplementary Figure 1**). The Ki67 positivity rate was 26%, suggesting high proliferative activity (**Figure 2F**). Ki67 positivity was particularly marked in the solid nest structures (**Figure 2F**).

**Figure 3:**
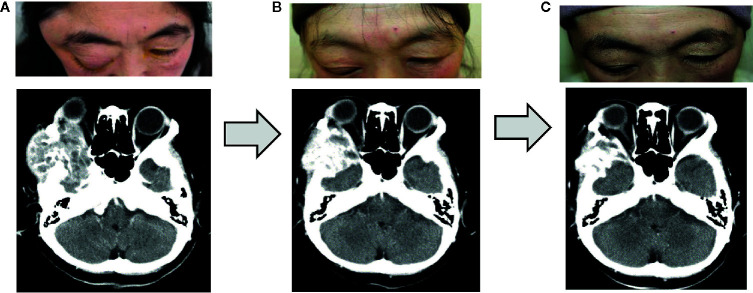
Changes after the treatment. Three courses of nab-paclitaxel chemotherapy and 3 months of treatment with Letrozole and Abemaciclib were performed, and the right orbital metastatic tumor shrank substantially. **(A)** Before chemotherapy. **(B)** After three courses of nab-paclitaxel. **(C)** Three months after treatment with Letrozole and Abemaciclib.

Because the metastatic orbital tumor compressed the cerebrum and was thus life-threatening, we immediately started broad-spectrum chemotherapy appropriate for the tumor. As there was severe damage to the corneal epithelium, a solution of purified sodium hyaluronate for ophthalmic instillation and ofloxacin ophthalmic ointment were administered. We performed three courses of nab-paclitaxel therapy (at a dose of 260 mg/m² by intravenous infusion at every three weeks). Because numbness and peripheral sensory neuropathy appeared as an adverse effect, we switched to combination therapy with Letrozole (an aromatase inhibitor: 2.5 mg/day per os) and Abemaciclib (a CDK4/6 inhibitor: 300 mg/day per os) for hormone receptor-positive, HER2-negative advanced breast cancer after three courses of nab-paclitaxel therapy, and this resulted in marked reduction of the orbital tumor (**Figures 3A–C**).

**Figure 4:**
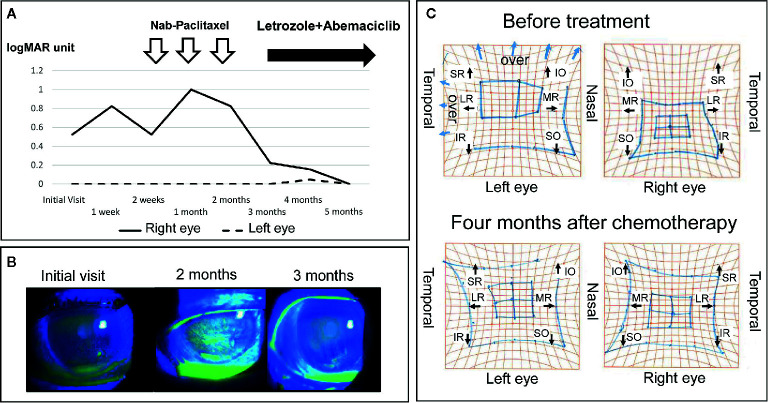
Changes in visual function, ocular surface disorders (fluorescein stain) and eye movement before and after treatment. **(A)** Right visual acuity improved to within the normal range. **(B)** Right ocular surface disorders such as superficial punctate keratopathy and erosion improved gradually. **(C)** A comparison of the Hess screen test before treatment and 4 months after the induction of chemotherapy showed a clear improvement in the movement restriction of the right eye. SR, superior rectus muscle; IO, inferior oblique muscle; LR, lateral rectus muscle; MR, medial rectus muscle; IR, inferior rectus muscle; SO, superior oblique muscle.

Hertel exophthalmometry showed marked improvement in her right eye; 19 mm at 4 months after the start of chemotherapy, and 17 mm at 5 months after the start of chemotherapy. Marked improvements were presented in upward mobility of the right eye, and in the results of Goldmann perimetry. The corneal epithelial disorders in the right eye also showed obvious improvement. These positive effects of the chemotherapy resulted in improvement of right visual acuity (**Figures 4A–C**). Two years after her initial visit, the patient’s general condition and quality of life are still good, and her breast cancer and orbital metastasis are still decreasing in size. She has been very satisfied with the treatment so far.

## Discussion

Intraocular metastasis is often a component of systemic metastasis in patients with cancer. It arises *via* hematogenous transport, passing through the choroid where blood flow is abundant (2). Metastatic orbital tumors are reported; breast cancer (53%), prostate cancer (12%), lung cancer (8%), melanoma (6%), and kidney cancer (5%) (2). Orbital metastasis from breast cancer is typically unilateral, appears 4.5 to 6.5 years after the treatment of the primary lesion, and locates in orbital fat tissue and extraocular muscles, from where it may invade the orbital bone. Among the histologic types of breast cancer, the frequency of ductal carcinoma is as high as 78%, whereas the frequency of lobular carcinoma is about 11% (3). However, lobular carcinoma accounts for the majority of orbital metastases from breast cancer. Orbital metastasis from ductal carcinoma of the breast has also been reported, but it is rare and case reports have been infrequent (4).

Typical subjective symptoms include diplopia, pain, ptosis, and objective findings include impaired eye movement, exophthalmos, and eye deviation (5). Those ocular symptoms were shown prior to primary lesion diagnosis about 25% of cases (5). In the present case, the predominant symptom was exophthalmos and foreign body sensation due to corneal damage. If ocular symptoms occur in a patient with a history of breast cancer, then orbital metastasis should be suspected, and ophthalmologic examinations such as visual field tests and diagnostic imaging should be performed.

In our search of Pubmed using breast cancer and orbital metastasis as keywords, we reviewed metastatic orbital tumors from breast cancer; no other organ metastasis, ocular symptoms as the chief compliant, and confirmation of histological diagnosis. We were able to extract 14 references (4, 6–18). They are listed in chronological order (**Supplementary Table 1**). The patients ranged in age from 30 to 83 years, and included 13 females and 1 male (**Supplementary Table 1**). Eight patients had a history of breast cancer and six had ocular symptoms prior to cancer diagnosis. Symptoms included diplopia, ocular pain, and ocular movement disorder. The histological types of the 14 previously reported breast cancers were lobular carcinoma in nine patients, ductal carcinoma in four, and mixed type in one. Neuroendocrine differentiation was not examined in any of the 14 cases. The main treatments employed were surgery, radiation therapy, and hormone therapy (**Supplementary Table 1**). Despite multidisciplinary treatment, most patients died within 2 to 3 years, and the present patient with a large orbital tumor appears to be the first reported to have a clear improvement in visual function after chemotherapy.

In this case, surgery was not applicable for her stage IV cancer. The giant orbital tumor compressed the cerebrum and chemotherapy with nanoparticle albumin-bound paclitaxel was started without waiting for the human epidermal growth factor receptor 2 (HER2) results and subtype details. Biopsy of the orbital region was not performed to avoid the dissemination of tumor cells. After three courses of chemotherapy followed by combination therapy, the orbital tumor decreased dramatically in size, and ocular symptoms improved considerably; the damage to the cornea, limited upward rotation of the eyeball, and visual field loss. The patient’s general condition has been good up to the time of writing, and the orbital tumor showed shrinkage with appropriate treatment. Using a collaborative approach involving various specialists, we have successfully diagnosed and treated patients with visual impairment that has had a direct effect on their quality of life.

Breast carcinoma with neuroendocrine differentiation is a rare histologic type of breast cancer that accounts for approximately 2%–5% of all cases (19). In the 4th edition of the World Health Organization (WHO) classification of tumors of the breast, neuroendocrine tumors were categorized into three types: well differentiated, poorly differentiated/small cell carcinoma, and invasive breast carcinoma with neuroendocrine differentiation (20). It is defined as uncommon subtype of breast cancer in which more than 50% of the tumor cells express at least one neuroendocrine marker (synaptophysin, chromogranin A, or neuron-specific enolase). The present case was histologically confirmed as ductal carcinoma with neuroendocrine differentiation, showing positivity for synaptophysin and focal positivity for neuron-specific enolase. ER/PR positivity and HER2 negativity were consistent with the characteristics of neuroendocrine type breast cancer. On the other hand, invasive breast cancer with neuroendocrine differentiation is not included in the neuroendocrine category in the 5th edition of the WHO classification, 2019 (21). However, it has been reported that invasive breast cancer with neuroendocrine differentiation is more likely to have worse disease free survival metastasize (22) and more aggressive characteristics than invasive breast cancer (23), and thus is considered a clinically important disease entity. In the present case, the Ki67 labeling index, which is an indicator of proliferation capacity, was as high as 26%, thus – along with neuroendocrine differentiation – possibly explaining the formation of the metastatic solitary giant orbital tumor.

CK is a cytoskeletal protein expressed mainly in epithelial cells, and more than 20 subtypes have been identified in various organs. The expression of CK7 and CK20 in metastases has been used to determine the organ in which the primary tumor has arisen. CK7 is expressed mainly in cancers of the breast, lung, and salivary gland, whereas CK20 is frequently expressed in cancers of the colon and pancreas. Breast cancer is generally CK7-positive and CK20-negative. This immunostaining feature supported the possibility that the metastasis from an unknown primary site had originated from breast cancer, although CK7-negative cancers have also been reported, occasionally. Decreased immunostaining for pan-cytokeratin has also been reported in breast cancer patients who are at high risk for metastasis (24). Moatamed et al. studied in detail CK7-negative breast cancers and reported that they were ductal carcinomas positive for estrogen receptor and negative for HER2, with a high Ki-67 grade and often neuroendocrine differentiation (25). Recently Lu et al. reported that the frequency of CK 7-negative breast cancer was higher in Grade 3 according to the Nottingham grade classification. It has also been reported that high proliferative placenta-derived multi-potent cells express CK 7 at low level (26). Moreover, it has been reported that loss of CK 7 leads to increased proliferation of the bladder urothelium in CK 7-knockout mice (27). Accordingly, the neuroendocrine differentiation and CK 7 negativity in the present case were considered to have been indicative of a high capacity for distant metastasis and proliferation, resulting in formation of a well-defined orbital mass.

The diagnosis of the metastatic orbital tumor should be performed by histologic examination of the orbit with open biopsy. Diagnostic yield and accuracy of the biopsy are depending on the size, site, timing, and the technique of procurement (28). In the present case, biopsy from the ophthalmic tumor site was difficult to perform because [1] the tumor was enlarged and associated with cerebral compression, [2] there was a risk of ocular damage, hemorrhage, and tumor dissemination, and [3] the patient did not consent to the biopsy. Multiparametric MRI showed that the orbital tumor was hypointense and hyperintense relative to fat on T1-weighted and T2-weighted images, respectively, as well as being contrast enhanced by gadolinium, thus showing the characteristics of a metastatic tumor. Both chemotherapy and combination therapy (hormonal therapy plus chemotherapy) for this invasive ductal cancer with endocrine differentiation (hormone receptor-positive, HER2-negative) was significantly effective against both the breast and orbital tumors. Although histological examination of the orbital tumor would have been desirable, on the basis of the collateral evidence, we considered the case to be a metastatic orbital tumor from the breast cancer. However, accumulation of further cases will be necessary to confirm the effectiveness of this approach.

In conclusion, we have described our experience of a case of ductal carcinoma with neuroendocrine differentiation metastatic to the orbit that responded well to chemotherapy, achieving tumor reduction and improvement of visual function. We consider that CK7-negative ductal carcinoma with neuroendocrine differentiation may have a high potential for distant metastasis, and that in such cases clinicians should be aware that orbital metastasis may be an initial symptom.

## Data Availability Statement

The original contributions presented in the study are included in the article/**Supplementary Material**. Further inquiries can be directed to the corresponding author.

## Ethics Statement

The studies involving human participants were reviewed and approved by the ethics committee of Yamagata University Faculty of Medicine. The patients/participants provided their written informed consent to participate in this study. Written informed consent was obtained from the individual(s) for the publication of any potentially identifiable images or data included in this article.

## Author Contributions

KT was involved in patient management, collected clinical data, reviewed the literature, and prepared the manuscript. KN and HY were responsible for study design and writing. KN, HN, and HY revised the manuscript. SH, SG, YT, SS YY, EK, TY, YS, and TS participated in the treatment of the patient. TK and MY performed pathological diagnosis. HY contributed to supervision of the study and made final approval of the version to be published. All authors contributed to data collection and interpretation, and clinically reviewed the manuscript. All authors contributed to the article and approved the submitted version.

## Funding

This work was supported by the Ministry of Education, Culture, Sports, Science and Technology of Japan (19K18870).

## Conflict of Interest

The authors declare that the research was conducted in the absence of any commercial or financial relationships that could be construed as a potential conflict of interest.
