# Supplementary figures and images for: Metastatic Orbital Tumor From Breast Ductal Carcinoma With Neuroendocrine Differentiation Initially Presenting as Ocular Symptoms: A Case Report and Literature Review
Source: Front Endocrinol (Lausanne). 2021 Feb 22;12:625663. doi: 10.3389/fendo.2021.625663 (PMC7937957; doi:10.3389/fendo.2021.625663)

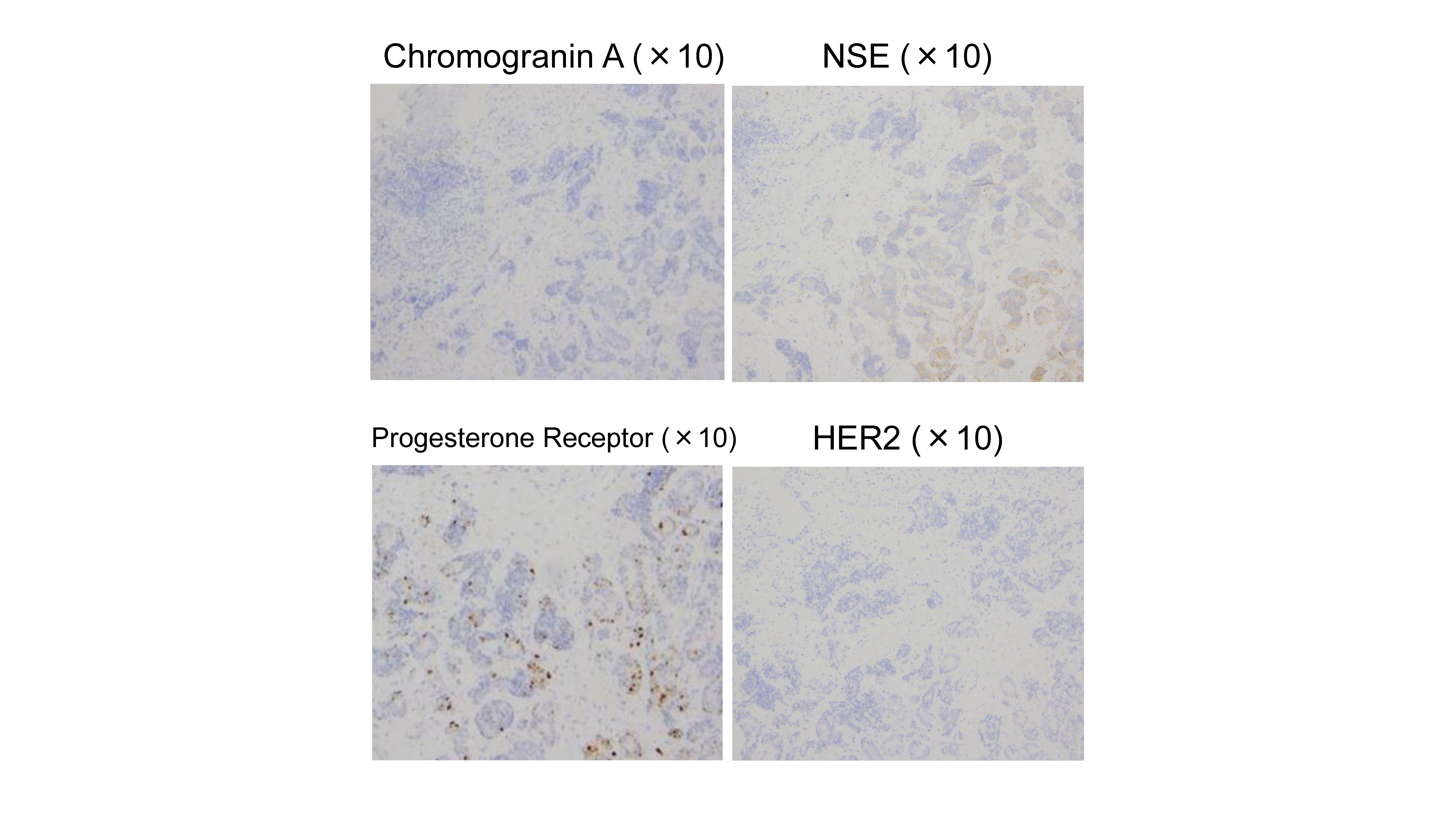

Supplement: Supplementary file 1 [file Image_1.jpeg]
