# Supplementary material for: Metastatic Orbital Tumor From Breast Ductal Carcinoma With Neuroendocrine Differentiation Initially Presenting as Ocular Symptoms: A Case Report and Literature Review
Source: Front Endocrinol (Lausanne). 2021 Feb 22;12:625663. doi: 10.3389/fendo.2021.625663 (PMC7937957; doi:10.3389/fendo.2021.625663)
Supplement: Supplementary file 2 [file Table_1.pdf]

Supplementary Table 1

Summary of case reports of orbital metastases from histologically confirmed breast cancers with early eye manifestations -Literature review-

First authors/s, year	Age	Sex	Early eye manifestation	Histology	IHC	Interval between primary diagnosis and orbital metastasis	Treatments	Outcomes	(Refs.)
Toller KK, et al, 1990	47	F	Bilateral orbital pain<br>Decreased vision<br>Binocular diplopia	LC	NA	8 years	Right anterior orbitotomy	Died after 9 months	6
Stuntz M, et al, 1998	40	M	Right eye pain<br>Decrased visual activity<br>Periorbital soft tissue swelling<br>Severe right eye proptosis<br>Corneal infection refractory to medical therapy	DC	NA	Initial manifestation	Radiation therapy<br>Chemotherapy<br>Hormonal therapy	Complete vision loss in the right eye<br>Died after 3 years	7
Lell M, et al, 2004	63	F	Swelling of both eyelids<br>Impaired eye movements	LC	CK7 (+)	Initial manifestation	Radical mastectomy	NS	8
Van der Zee J, et al, 2004	83	F	Tumor palpitation	LC	NA	1.5 years	Re-radiation<br>Hyperthermia<br>Surgery<br>Hormonal therapy	Tumor regression<br>Died 24 months after treatment	9
Solari HP, et al, 2006	81	F	Unilateral painless red eye<br>Proptosis<br>A nodule in the superior orbital rim	DC	ER(+)	2 years	Radiation therapy	NS	10

Supplementary Table 1 Continued

First authors/s, year	Age	Sex	Early eye manifestation	Histology	IHC	Interval between primary diagnosis and orbital metastasis	Treatments	Outcomes	(Refs.)
Kadivar M, et al, 2006	53	F	Left orbital pain<br>Mild proptosis	LC	ER(+)	Initial manifestation	NS	NS	11
Talwar V, et al, 2007	30	F	Protrusion of right eyeball<br>Swelling around the right eye	DC	ER(+)<br>PR(+)	1.5 years	Right lateral orbitotomy<br>Radiation therapy<br>Tamoxifen	No evidence of local recurrence	12
Surace D, et al, 2008	73	F	Left palpebral ptosis<br>Diplopia<br>An inability to close the eylids<br>Severe limination of eye movement	LC	ER(+)	25 years	Hormonal therapy	Modest improvement	13
Gupta S, et al, 2011	46	F	Left orbital pain	LC	ER(+)<br>PR(+)<br>HER2(-)	Initial manifestation	Bilateral mastectomy<br>Resection of left orbital mass<br>Tamoxifen and leuprolide	Disease free	14
Tomizawa Y, et al, 2012	70	F	Worsening blurred vision<br>Periorbital swelling<br>Redness in the right eye	LC	ER(+)<br>CK(+)<br>HER2(-)	Initial manifestation	Hormonal therapy	NS	15
Patel MM, et al, 2013	47	F	Transient monocular vision loss	LC	ER(+)<br>PR(-)<br>HER2(-)	1.5 years	Radiation therapy<br>Orbitotomy	NS	16

Supplementary Table 1 Continued

First authors/s, year	Age	Sex	Early eye manifestation	Histology	IHC	Interval between primary diagnosis and orbital metastasis	Treatments	Outcomes	(Refs.)
Saffra N, et al, 2014	46	F	Diplopia<br>Pain in the left eye	LC	ER(+)<br>PR(+)<br>HER2(-)	Initial manifestation	Tamoxifen and leuprolide<br>Mastectomy	Complete resolution of the ocular symptoms	17
Gondim DD, et al, 2017	80	F	Diplopia	LC	ER(+)<br>PR(-)<br>CK7(+)<br>CK(20(-)	NS	NS	NS	18
Framarino-Dei-Malatesta M, et al, 2019	51	F	Diplopia<br>Blurred visoin<br>Upper movement restriction of right eye	DC	ER(+)<br>PR(+)<br>HER2(-)<br>Ki67 (35%)	7 years	Radiation therapy plus fulvestrant<br>Chemotherapy (palbociclib)<br>Anti-hormone based therapy	Failed to improve eye symptom	4
**Present Case**	50's	F	Foreign body sensation<br>Exophthalmos	DC	ER(+)<br>PR(+)<br>Ki67 (26%)<br>SYP(+)<br>CK7(-)<br>CK20(-)	Initial manifestation	Chemotherapy<br>Nab-paclitaxel<br>Letrozole and abemaciclib	Alive	

IHC: immunohistochemistry; NA: data not available; NS: not specified; LC: lobular carcinoma; DC: ductal carcinoma; ER: estrogen receptor; PR: progesterone receptor; HER2: human epidermal growth factor receptor 2; CK: cytokeratin
